# Supplementary figures and images for: Estimating the distribution of parameters in differential equations with repeated cross-sectional data
Source: PLoS Comput Biol. 2024 Dec 23;20(12):e1012696. doi: 10.1371/journal.pcbi.1012696 (PMC11706453; doi:10.1371/journal.pcbi.1012696)

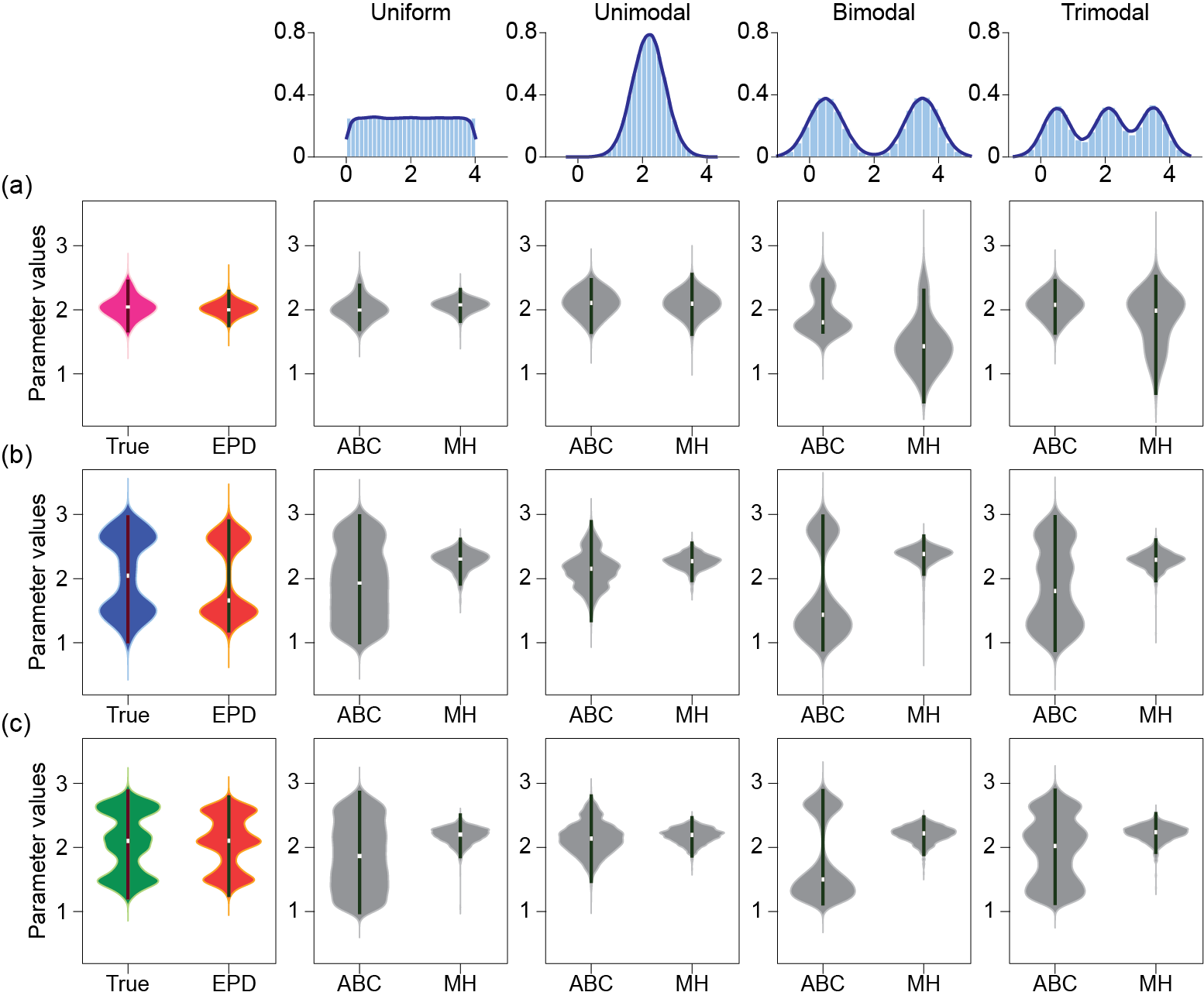

Supplement: S1 Fig — We estimate three True parameter distributions (unimodal (True-red), bimodal (True-blue), and trimodal (True-green)) corresponding to each dataset in Fig 1. Without any assumptions about the prior distribution, EPD can accurately estimate the True parameter distributions (EPD-orange). (a-c). When the True parameter distribution is unimodal, all estimation results closely approximate the true distribution, except for ABC with a bimodal prior distribution (a). However, when the true distribution is bimodal, all estimation results using ABC and MH show different patterns from the true distribution, except for ABC with a bimodal prior distribution (b). Similar to (b), when the true distribution is trimodal, all estimation results using ABC and MH show different patterns from the true distribution, except for ABC with a trimodal prior distribution (c). These results indicate that the ABC method estimates the similar shape of parameter distribution to the prior distribution, leading to inaccurate parameter estimates with inaccurate choice of prior distributions. (PNG) [file pcbi.1012696.s001.png]

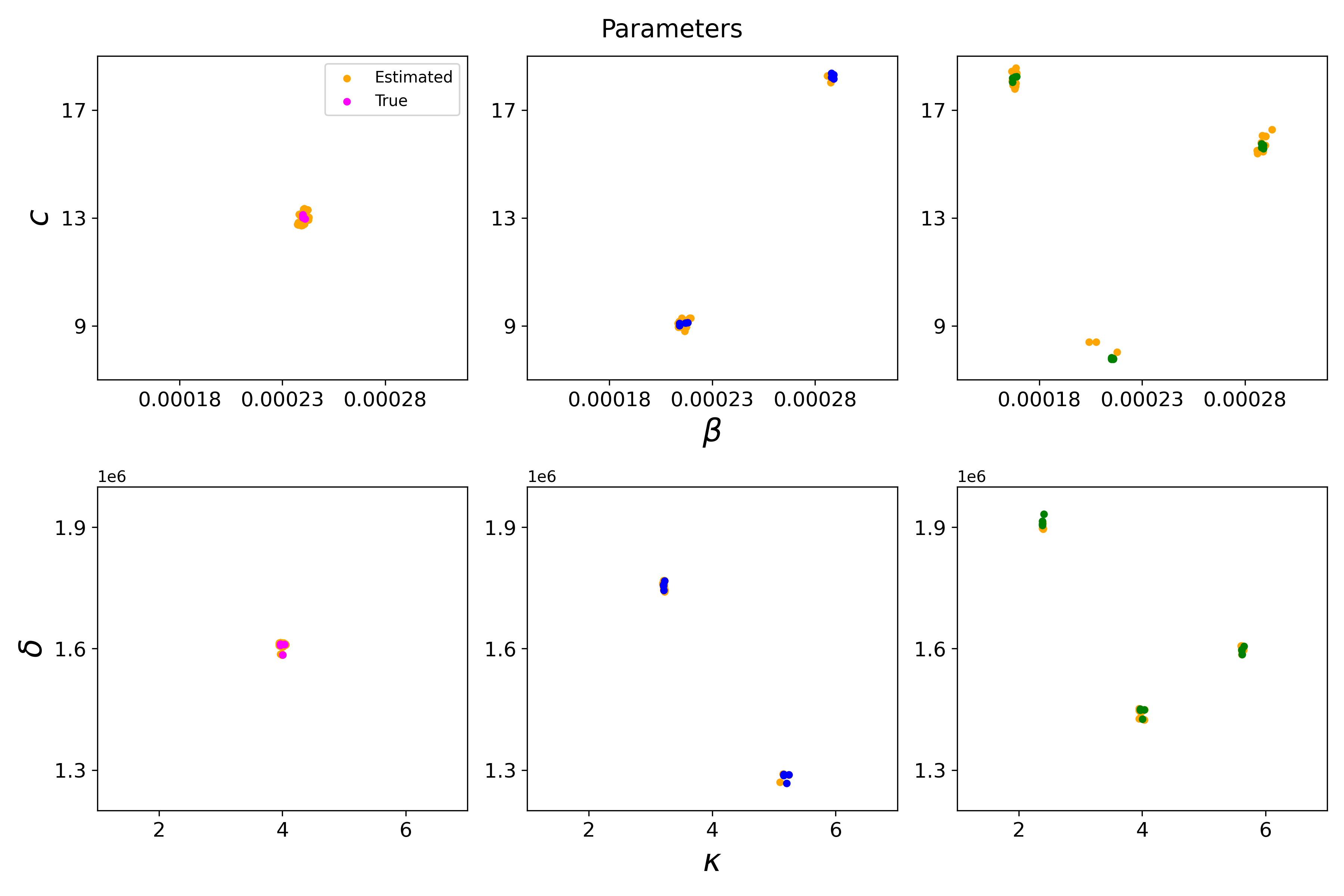

Supplement: S2 Fig — We estimated the distributions of β, c, κ, and δ, corresponding to data in (Fig 5, left T(t) − V(t)), respectively. First, we applied EPD to data generated from parameters that share similar scales (Fig 5(a), left T(t) − V(t)). Consequently, EPD can accurately estimate the parameters (Left). Furthermore, with data generated from parameters with different scales (Fig 5(b) and 5(c), left), EPD can infer the true parameter distributions (Middle and Right, respectively). That is, EPD can estimate the true distribution of parameters even when they do not follow the normal distribution. Notably, the prediction does not contain the interpolation of the centers as not previously in the logistic model. (PNG) [file pcbi.1012696.s002.png]

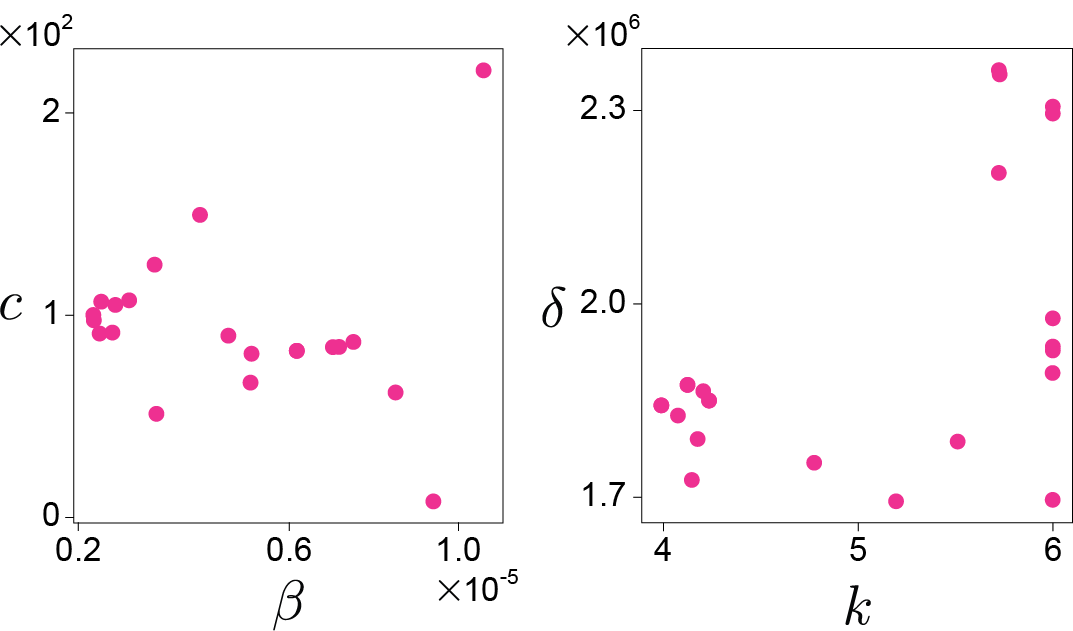

Supplement: S3 Fig — We estimated the four parameters β, c, κ, δ that fit the target cell-limited model to the given RCS data [25]. We discovered that the estimated parameter distributions contain heterogeneity for all parameters, similar to the estimates for p and Kδ. Unlike previous results in [15], our findings do not follow the normal distribution shape. Nevertheless, these predictions could be a reasonable guess because they can reconstruct the trajectories through Eq 1. (PNG) [file pcbi.1012696.s003.png]

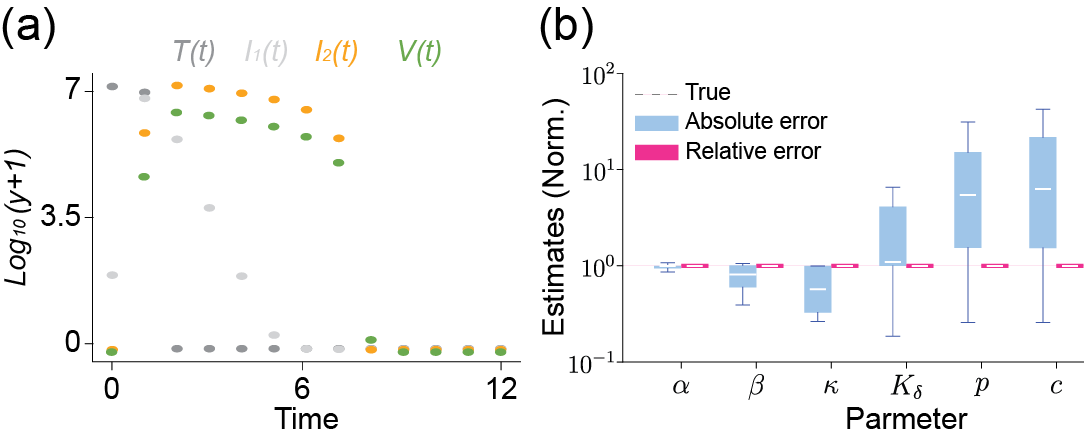

Supplement: S4 Fig — (a) We generated trajectories for T(t), I1(t), I2(t), and V(t) corresponding to one set of six parameters (p, Kδ, β, c, κ, δ) = (2.4 × 10^−4^, 1.6, 13.0, 4.0, 1.6 × 10^6^, 4.5 × 10^5^) within the target cell-limited model. (b) We compared the parameter estimates obtained using the absolute error (i.e., Ln(p)=∑i=1T|y¯n(ti;p)-y˜n(ti)|2), to those obtained using the relative error Ln(p) in (Eq 4) (Blue and red box plots, respectively). The box plot results reveal that the absolute error introduces biases in the parameter estimates compared to the relative error. (PNG) [file pcbi.1012696.s004.png]

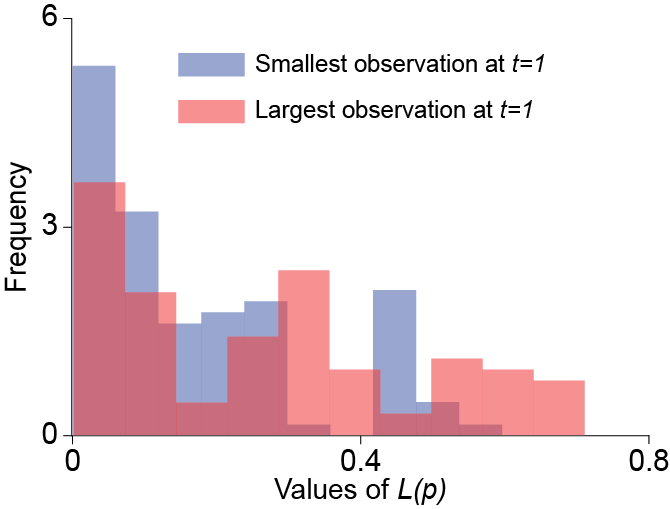

Supplement: S5 Fig — The distribution of L(p) varies according to the magnitude of the observations. As the L(p) distribution associated with the larger observation tends to have higher overall values, the likelihood of it being ultimately selected decreases. (PNG) [file pcbi.1012696.s005.png]

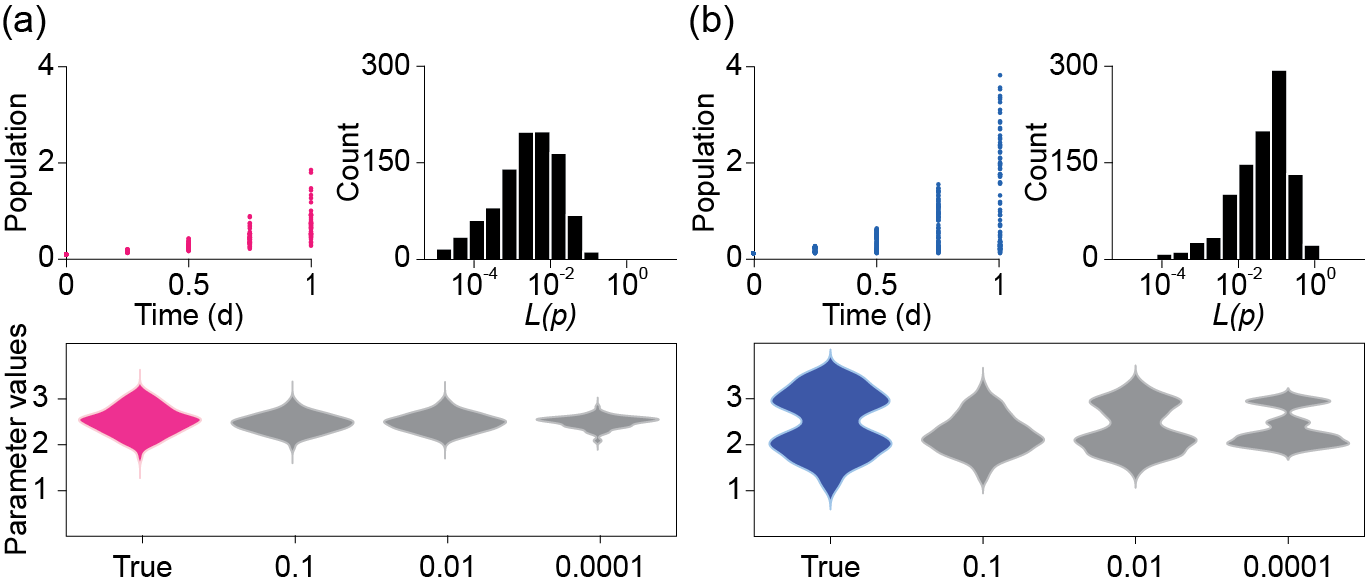

Supplement: S6 Fig — (a) When the shape of the underlying distribution is unimodal (True), L(p) with relative error in EPD estimation is not significantly affected by the threshold values (0.1, 0.01, 0.0001). (b) Conversely, if the underlying distribution is bimodal (True), the estimated shape can differ from the underlying distribution depending on the threshold values. (PNG) [file pcbi.1012696.s006.png]

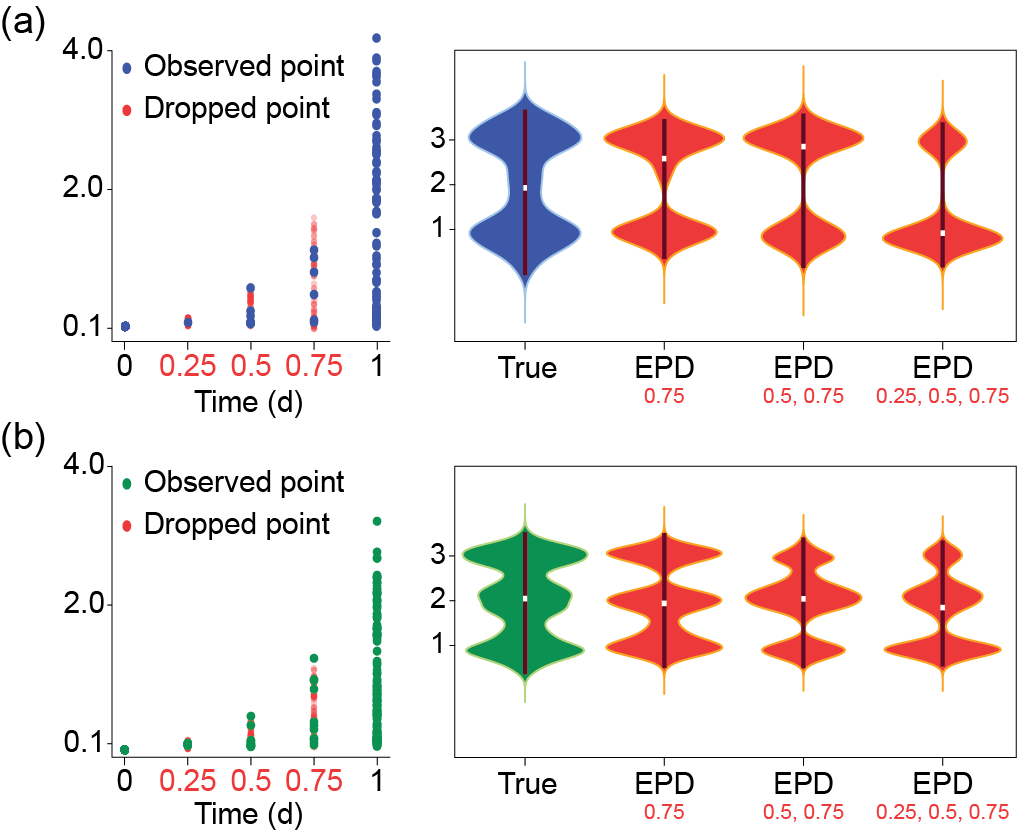

Supplement: S7 Fig — (a) When the underlying parameter distribution is bimodal (right, True), EPD accurately estimates the True parameter distribution (EPD 0.75) using RCS data after removing 90% of the observation data points at t = 0.75. Similarly, when 90% is removed at t = 0.5 and t = 0.75, the estimation using EPD remains close to the underlying distribution (EPD 0.5, 0.75). However, when 90% is removed at t = 0.25, t = 0.5, and t = 0.75, a bias is observed in the height of the peak (EPD 0.25, 0.5, 0.75). (b) When the underlying parameter distribution is trimodal (right, True), EPD can estimate the underlying distribution using RCS data after removing 90% of the data points at t = 0.75 (right, EPD 0.75). Unlike the bimodal case (a), the peak heights differ from the True distribution in two cases: 1) when observation data points are removed at t = 0.5 and t = 0.75 (right, EPD 0.5, 0.75), and 2) when points are removed at t = 0.25, t = 0.5, and t = 0.75 (right, EPD 0.25, 0.5, 0.75). Notably, although the missing data yields the bias in the heights, EPD can still estimate the shape of the parameter distribution, stemming from the heterogeneity of the data. (PNG) [file pcbi.1012696.s007.png]
