# Supplementary material for: Estimating the distribution of parameters in differential equations with repeated cross-sectional data
Source: PLoS Comput Biol. 2024 Dec 23;20(12):e1012696. doi: 10.1371/journal.pcbi.1012696 (PMC11706453; doi:10.1371/journal.pcbi.1012696)
Supplement: S1 Algorithm — (PDF) [file pcbi.1012696.s008.pdf]

S1 Algorithm: Approximate Bayesian Computation (ABC)

Input: Observed data $Y = \{Y_i\}_{i=1}^{T}$, prior distribution $D(\mathbf{p})$, tolerance level ϵ, number of samples N

Output: Posterior distribution: a set of accepted samples $A \subseteq \{\mathbf{p}_n\}_{n=1}^{N}$

Initialize the set of accepted parameters $A \leftarrow \emptyset$

For $n = 1$ to N do

Sample parameter $\mathbf{p}_n \sim D(\mathbf{p})$

Solve Eq. (2) using $\mathbf{p}_n$ to obtain $\bar{y}_n(t; \mathbf{p}_n)$

Compute the distance $d(Y, \mathbf{p}_n) = \sum_{i=1}^{T} \min_{1 \le j \le J_i} \left| y_j(t_i) - \bar{y}_n(t_i; \mathbf{p}_n) \right|$

If $d(Y, p_n) \le \epsilon$, then

$A \leftarrow A \cup \{\mathbf{p}_n\}$ ▷ Add the parameter if the distance is within tolerance

End if

End for

Return: Set of accepted parameters A as the approximate posterior distribution
