# Supplementary material for: Estimating the distribution of parameters in differential equations with repeated cross-sectional data
Source: PLoS Comput Biol. 2024 Dec 23;20(12):e1012696. doi: 10.1371/journal.pcbi.1012696 (PMC11706453; doi:10.1371/journal.pcbi.1012696)
Supplement: S2 Algorithm — (PDF) [file pcbi.1012696.s009.pdf]

**S2 Algorithm: Metropolis-Hastings Algorithm for Bayesian Inference**

**Input:** Observed data $Y = \{Y_i\}_{i=1}^{T}$, prior distribution $D(\mathbf{p})$, likelihood function $L(\mathbf{y}|\mathbf{p})$, proposal distribution $q(\mathbf{p}'|\mathbf{p})$, initial value $\mathbf{p}_0$, number of iterations $N$.

**Output:** Sequence of posterior samples $\{\mathbf{p}_n\}_{n=1}^{N}$

Initialize $\mathbf{p}_0$

**For** $n = 1$ to $N$ do

Select a new candidate $\mathbf{p}'$ from $q(\mathbf{p}'|\mathbf{p}_{n-1})$

Compute the acceptance probability:

$$\alpha = \min\left(1, \frac{\mathbb{P}(\mathbf{p}'|Y)q(\mathbf{p}_{n-1}|\mathbf{p}')}{\mathbb{P}(\mathbf{p}_{n-1}|Y)q(\mathbf{p}'|\mathbf{p}_{n-1})}\right)$$

$$= \min\left(1, \frac{L(Y|\mathbf{p}')D(\mathbf{p}')q(\mathbf{p}_{n-1}|\mathbf{p}')}{L(Y|\mathbf{p}_{n-1})D(\mathbf{p}_{n-1})q(\mathbf{p}'|\mathbf{p}_{n-1})}\right)$$

$$= \min\left(1, \Pi_{i=1}^{T}\Pi_{j=1}^{J_i}\frac{L\big(y_j(t_i)|\mathbf{p}'\big)D(\mathbf{p}')q(\mathbf{p}_{n-1}|\mathbf{p}')}{L\big(y_j(t_i)\big|\mathbf{p}_{n-1}\big)D(\mathbf{p}_{n-1})q(\mathbf{p}'|\mathbf{p}_{n-1})}\right),$$

Where $L\big(y_j(t_i)\big|\mathbf{p}\big) = \frac{1}{\sqrt{2\pi}\sigma}\exp\left(-\frac{\big(y_j(t_i)-y(t_i;\mathbf{p})\big)^2}{2\sigma^2}\right)$ (Recall $\bar{y}(t;\mathbf{p})$ for eq. (2))

Sample $u \sim$ Uniform(0, 1)

**If** $u \leq \alpha$, then

$\mathbf{p_n} = \mathbf{p}'$

**Else**

$\mathbf{p_n} = \mathbf{p_{n-1}}$

**End if**

**End for**

**Return:** Sequence of posterior samples $\{\mathbf{p}_n\}_{n=1}^{N}$
