# Supplementary material for: Estimating the distribution of parameters in differential equations with repeated cross-sectional data
Source: PLoS Comput Biol. 2024 Dec 23;20(12):e1012696. doi: 10.1371/journal.pcbi.1012696 (PMC11706453; doi:10.1371/journal.pcbi.1012696)
Supplement: Attachment — Submitted filename: EPD_PLOS_comp_bio_Review response.pdf [file pcbi.1012696.s010.pdf]

Review Report on PCOMPBIOL-D-24-00903, “Estimating the Distribution of Parameters in Differential Equations with Repeated Cross-Sectional Data”

We thank the reviewers for their constructive comments and suggestions for strengthening the presentation of our manuscript. In response, we have revised the manuscript. Below, we give our detailed responses to the reviewers’ comments. The reviewers’ comments appear in Blue, and our responses in Black (italics indicate the revised text of the manuscript).

Reviewer comments:

Reviewer #1: The manuscript proposes a novel parameter estimation method for when the given observations are repeated cross-sectional (RCS) data. The problem of model fitting using RCS is prevalent in biomedical research, and hence the manuscript addresses an important methodological problem. The proposed model is presented without a rigorous theoretical justification, but the authors provide a wide range of examples and simulation studies to demonstrate that their methods work well for common mathematical biology problems. I have identified only a few minor issues to raise:

1. The authors could use some copy editing to improve the writing. Currently, there are too many awkward sentences throughout the manuscript.

Answer: In response to the reviewer’s comments, we have carefully reviewed the manuscript and the supplemental materials.

2. The presented method is very similar to the approximate Bayesian computation (ABC) approach. Proper citations to the literature on ABC would be useful.

Answer: We thank the reviewer for pointing it out. We have cited the ABC approach [marjoram2003markov, cranmer2020frontier] in Introduction (line 33). As reviewer’s comment, our method is indeed motivated by ABC (we added this in line 39 through this revision).. However, it is theoretically known that Baysian-based estimation (such as ABC, Metropolis-Hastings (MH)) of parameter distribution can vary depending on the shape of prior distribution [swigon2019importance]. Because we are dealing with RCS data, the prior dependency is inevitably stronger. To demonstrate this, we have included the estimation results using ABC and MH with different prior shapes in the exponential model. (Figure S1):

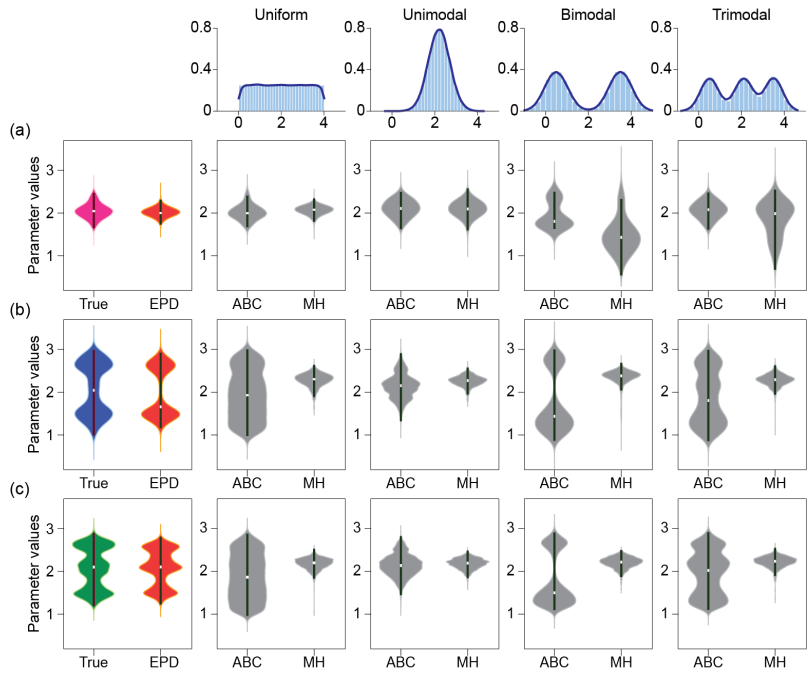

Fig S1. Parameter estimation results for the Exponential model using Approximate Bayesian Computation (ABC) and Metropolis-Hastings (MH), based on different shapes of the prior distribution (uniform, unimodal, bimodal, and trimodal). We estimate three True parameter distributions (unimodal (True-red), bimodal (True-blue), and trimodal (True-green)) corresponding to each dataset in Fig. 1. Without any assumptions about the prior distribution, EPD can accurately estimate the True parameter distributions (EPD-orange). (a-c). When the True parameter distribution is unimodal, all estimation results closely approximate the true distribution, except for ABC with a bimodal prior distribution (a). However, when the true distribution is bimodal, all estimation results using ABC and MH show different patterns from the true distribution, except for ABC with a bimodal prior distribution (b). Similar to (b), when the true distribution is trimodal, all estimation results using ABC and MH show different patterns from the true distribution, except for ABC with a trimodal prior distribution (c). These results indicate that the ABC method estimates the similar shape of parameter distribution to the prior distribution, leading to inaccurate parameter estimates with inaccurate choice of prior distributions.

To avoid dependency on the prior distribution, EPD can automatically derive the prior distribution from the observed data. The acceptance probability was also determined using the data to remove researcher's subjectivity. We also have added this explanation in the Introduction and Method sections of the manuscript (line 45 and 343). We sincerely appreciate the reviewer for pointing out this important aspect.

References

[marjoram2003markov] Marjoram, P., Molitor, J., Plagnol, V., & Tavaré, S. (2003). Markov chain Monte Carlo without likelihoods. Proceedings of the National Academy of Sciences, 100(26), 15324-15328.
[cranmer2020frontier] Cranmer, K., Brehmer, J., & Louppe, G. (2020). The frontier of simulation-based inference. Proceedings of the National Academy of Sciences, 117(48), 30055-30062.
[swigon2019importance] Swigon, D., Stanhope, S. R., Zenker, S., & Rubin, J. E. (2019). On the importance of the Jacobian determinant in parameter inference for random parameter and random measurement error models. SIAM/ASA Journal on Uncertainty Quantification, 7(3), 975-1006.

3. In Figures 3b and 3c, while the different modes are correctly identified by the proposed algorithm, the weights for the modes differ from the true weights. For example, in Figure 3c, EPD puts more weight on the parameter value of 2 even when there is no noise. Some comments or analysis on why this happens are needed.

Answer: We found one reason that the bias was derived from the value of $L(\mathbf{p})$ in the EPD algorithm, measuring the discrepancy between the solution and the time series data. To check this, we divided $L(\mathbf{p})$ values obtained from Fig. 3(b)-observation with a sum of squares error into two groups: one group (blue) is selected from the smallest observation at the final time point ($t = 1$) and the other group (red) is selected from the largest observation. With two groups, we plotted a histogram (Fig. S5):

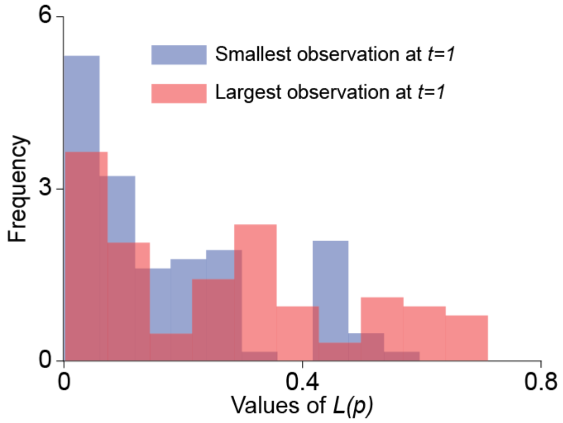

Fig S5. Distribution of $L(p)$ with an absolute error when a single observation is randomly sampled at each time point, with the smallest observation (blue) and the largest observation (red) selected at $t = 1$. The distribution of $L(p)$ varies according to the magnitude of the observations. As the $L(p)$ distribution associated with the larger observation tends to have higher overall values, the likelihood of it being ultimately selected decreases.

As a result, the parameter estimates from the red graph, which have relatively larger values of $L(\mathbf{p})$, exhibit relatively higher rejection probabilities, leading to a reduction in the magnitude of the corresponding peak in the estimated parameter distribution. We also observed that this bias still arises when using the absolute error with the log transformation. To avoid this bias, we have changed the measurement in $L(\mathbf{p})$ to the "relative" error. When $L(\mathbf{p})$ is defined as the relative error (Eq (3)), the estimation results in the exponential model indicate a similar level of peaks (Fig 3, right-EPD). In other words, by removing the effect of relative magnitude of observation points, we confirmed that the two parameters are maintained with similar likelihoods.

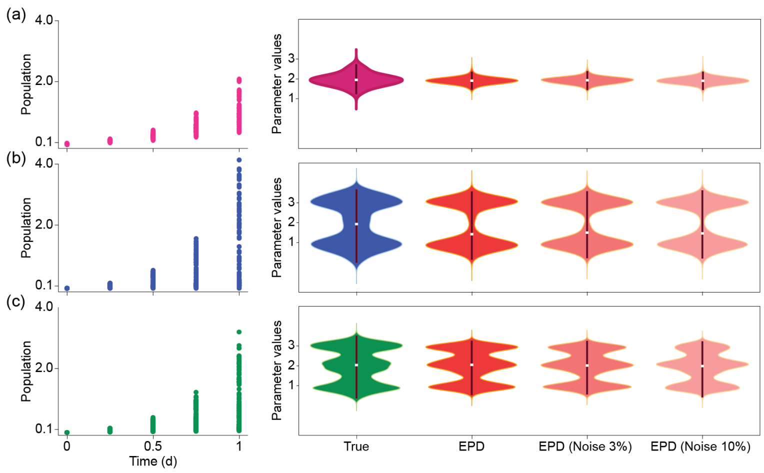

Fig 3 (revised) Estimation of true distributions by EPD in datasets exhibiting unimodal, bimodal, and trimodal parameter distributions within the exponential growth model. (a) When the true parameter distribution is unimodal, we applied EPD on the observed data (left) and estimated the parameters (right). Notably,

EPD accurately estimated parameter distributions even when we added 3% or 10% multiplicative noise to the data (b, c). Likewise, EPD was confirmed to estimate bimodal and trimodal parameter distributions effectively.

We sincerely thank the reviewer for pointing this out. We have noted the changes in the definition of $L(\mathbf{p})$ in the Materials and Methods section (line 334) and explained the cause of the bias issue in the Discussion section (line 244).

4. When discussing GP-based calibration, please include appropriate references such as Kennedy and O'Hagan (2001) in JRSSB.

Answer: We have added the suggested reference [kennedy2001bayesian] in line 25!

References

[kennedy2001bayesian] Kennedy, M. C., & O'Hagan, A. (2001). Bayesian calibration of computer models. Journal of the Royal Statistical Society: Series B (Statistical Methodology), 63(3), 425-464.

Reviewer #2: This paper introduces a novel method for parameter estimation in differential equations using repeated cross-sectional (RCS) data. Traditional methods for parameter estimation in differential equations often lead to data information loss, especially when dealing with RCS data. The authors present the Estimation of Parameter Distribution (EPD) method, which aims to accurately capture the distribution of parameters without losing data information. EPD involves generating synthetic time trajectories from RCS data, estimating parameters by minimizing the discrepancy between these trajectories and the true solution of the differential equation, and selecting parameters based on the scale of discrepancy. The authors evaluate EPD's performance across several models and apply it to real-world datasets, showing its advantage in capturing the shape of parameter distributions and addressing heterogeneity within systems.

The introduction of the EPD method seems novel and addresses the limitations of traditional parameter estimation methods. By focusing on RCS data, the authors present a solution that retains data information and estimates parameter distributions. The manuscript evaluates the EPD method across a few models, including exponential growth, logistic population models, and target cell-limited models with delayed virus production. The application of EPD to a few real-world datasets, such as amyloid beta accumulation and viral load, shows the practical utility of the method. The manuscript is also well-organized, and the results are clearly presented.

Several points could be worth including for elaboration and discussion:

1. The EPD method involves generating a large number of synthetic trajectories and estimating parameters for each, which can be computationally intensive. The manuscript does not provide a detailed discussion of the computational cost and potential strategies to mitigate it.

Answer: The computational cost is proportional to both the complexity of the underlying dynamical system and the number of artificial trajectories (line 328). To check this, we have added the details on computational cost table in the Materials and Methods section (p 17):

<table>
<tr><th>Equation</th><th>Parameter</th><th>n_y</th><th>n_p</th><th>T</th><th>J_i</th><th>N</th><th>Running Time (sec)</th></tr>
<tr><td rowspan="3">Exponential model</td><td>Unimodal</td><td rowspan="3">1</td><td rowspan="3">1</td><td rowspan="3">5</td><td>50</td><td rowspan="3">1000</td><td>4.06</td></tr>
<tr><td>Bimodal</td><td>100</td><td>4.18</td></tr>
<tr><td>Trimodal</td><td>150</td><td>4.26</td></tr>
<tr><td rowspan="3">Exponential Model w noise</td><td>Unimodal</td><td rowspan="3">1</td><td rowspan="3">1</td><td rowspan="3">5</td><td>50</td><td rowspan="3">10000</td><td>80.75</td></tr>
<tr><td>Bimodal</td><td>100</td><td>83.62</td></tr>
<tr><td>Trimodal</td><td>150</td><td>84.18</td></tr>
<tr><td rowspan="2">Logistic Model</td><td>Unimodal</td><td rowspan="2">1</td><td rowspan="2">2</td><td rowspan="2">5</td><td>12</td><td rowspan="2">3000</td><td>162.70</td></tr>
<tr><td>Bimodal</td><td>12</td><td>181.54</td></tr>
</table>

	Trimodal				12		202.69
	Real-world				12-13		188.85
Target Cell Limited model	Unimodal	4	6	13	12	1000	4555.18
	Bimodal				12		9036.38
	Trimodal				12		12318.30
	Real-World				10	10000	111667.74
Lorentz System	Unimodal	3	3	6	12	1000	28.90
	Bimodal				24		39.09
	Trimodal				36		42.62
Michaelis -Menten	Unimodal	2	2	6	12	1000	9.50
	Bimodal				24		11.39
	Trimodal				36		10.80

Table 3. Computational Cost for the EPD corresponding to each equation and dataset. The running time is measured based on several factors, including the number of equations (n_y), the number of parameters (n_p), the number of time points (T), the number of data points for each time point, (J_i), the total number of data points (N), and the number of artificial trajectories (N). The results indicate that the computational cost is directly proportional to n_y, n_p, T, and N, while showing a small correlation with the underlying parameter distribution.

As the reviewer pointed out, the computational costs for each artificial trajectory primarily increase due to the following two tasks: 1) solving the system using a numerical solver, and 2) fitting the solution through optimization techniques. For the first task, the computational time to generate the artificial trajectory is proportional to the number of equations. For the second task, as the number of parameters (or the dimension of the parameter space) increases, we need to explore a broader range of parameter space for each artificial trajectory. Consequently, the computational cost increases proportional to the number of equations, parameters, and artificial trajectories.

To overcome this, as a future direction, we are considering the use of a machine learning-based model emulator (such as Physics-informed Neural Networks incorporating hypernetworks [de2021hyperpinn] or DeepONet [lu2021learning, hadorn2022shift, seidman2022nomad, lee2023hyperdeeponet]) to handle larger systems with RCS data more efficiently. These emulators are pre-trained to approximate system solutions and parameters simultaneously, we can expect to significantly reduce the computational costs associated with these tasks.

We also have added the following limitation and future strategy to the Discussion section (line 277):

"...computational cost arises from when calculating the solution through a numerical solver and when fitting the solution through optimization techniques. As parameters were selected from a large set of synthetic trajectories that were obtained from the numerical solver, the computational costs became substantial, since it is proportional to both the complexity of equations and the number of trajectories. For future research, we are considering the use of a machine learning-based model emulator (such as Physics-informed Neural Networks incorporating hypernetworks [de2021hyperpinn] or DeepONet [lu2021learning, hadorn2022shift, seidman2022nomad, lee2023hyperdeeponet]) to handle larger systems with RCS data more efficiently. Specifically, these emulators, which are pre-trained to approximate system solutions and parameters simultaneously, we can expect to significantly reduce the computational costs associated with these tasks."

references

[de2021hyperpinn] de Avila Belbute-Peres, F., Chen, Y. F., & Sha, F. (2021). HyperPINN: Learning parameterized differential equations with physics-informed hypernetworks. The symbiosis of deep learning and differential equations, 690.
[lu2021learning] Lu, L., Jin, P., Pang, G., Zhang, Z., & Karniadakis, G. E. (2021). Learning nonlinear operators via DeepONet based on the universal approximation theorem of operators. Nature machine intelligence, 3(3), 218-229.
[hadorn2022shift] Hadorn, P. S. (2022, March). Shift-deeponet: Extending deep operator networks for discontinuous output functions. ETH Zurich, Seminar for Applied Mathematics.
[seidman2022nomad] Seidman, J., Kissas, G., Perdikaris, P., & Pappas, G. J. (2022). NOMAD: Nonlinear manifold decoders for operator learning. Advances in Neural Information Processing Systems, 35, 5601-5613.
[lee2023hyperdeeponet] Lee, J. Y., Cho, S. W., & Hwang, H. J. (2023). HyperDeepONet: learning operator with complex target function space using the limited resources via hypernetwork. arXiv preprint arXiv:2312.15949.

2. The determination of the scaling factor CC in the accept probability function seems critical for the EPD method's accuracy. However, the manuscript did not provide an analysis of how different values of CC impact the results.

Answer: We have provided the detailed values of CC (Table 2) used in this study in Table 2 (line 364):

Type	Exponential	Logistic	Target Cell Limited Model	Lorentz	Michaelis Menten
Unimodal	200	100	300	100	300
Bimodal	200	100	300	100	300
Trimodal	200	200	300	300	300
Real	-	200	300	-	-

Table 2. Scaling factors for the EPD corresponding to each equation and dataset. The scaling factor C was selected within the range of 100 to 300 across the five different types of equation, to estimate distribution that closely matches the true distribution. For the real-world dataset, the scaling factor was determined based on the most prevalent value observed in the simulation datasets.

There is no strict guideline for selecting these values. One promising approach is to generate simulation data with a range similar to the actual data across various distributions in advance, and then determine the appropriate range of CC values that can estimate the distribution. While we also highlighted the importance of choosing the CC values (Fig. 10), we only selected CC values within the range of 100 to 300 across the five different types of systems.

We have included an explanation for the selection of CC in the acceptance probability in the Discussion section (line 262).

"... Although careful selection of the scaling factor is required, EPD can accurately estimate the underlying distributions across various differential equations using scaling factor values between 100 to 300 (See also Table 2)."

3. The authors mention the use of a logistic transformation in the accept probability calculation. The manuscript could benefit from a discussion on alternative transformations and their potential effects on the parameter estimation process.

Answer: The EPD was developed inspired by the Approximate Bayesian Computation (ABC) method. Therefore, in the early stages of development, we attempted to directly accept only the parameter estimates $\mathbf{p}$ where the corresponding $L(\mathbf{p})$, defined in Eq. (3), was below the threshold (i.e., we accept parameter $\mathbf{p}$ if $L(\mathbf{p}) < \varepsilon$). However, the shape of the parameter estimates varied depending on the value of ε (see Fig S6 below).

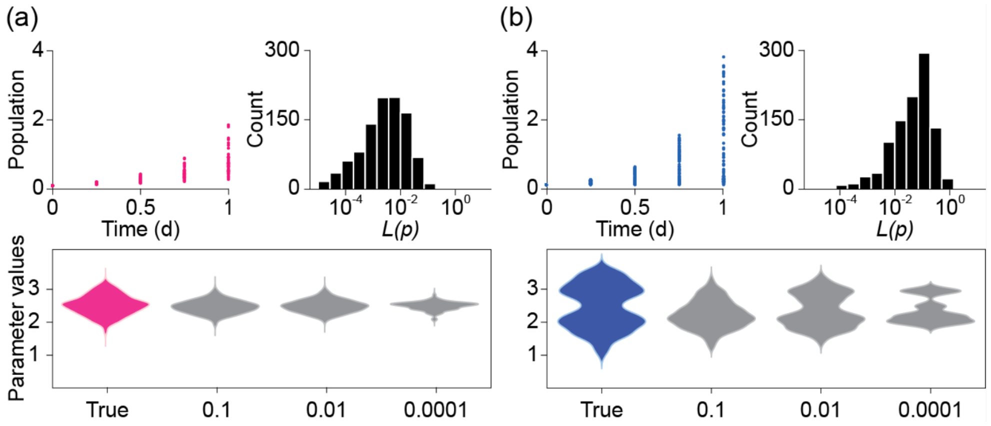

Fig S6. Estimation of the underlying parameter distribution of the Exponential model using EPD with relative error. (a) When the shape of the underlying distribution is unimodal (True), $L(\mathbf{p})$ with relative error in EPD estimation is not significantly affected by the threshold values (0.1, 0.01, 0.0001). (b) Conversely, if the underlying distribution is bimodal (True), the estimated shape can differ from the underlying distribution depending on the threshold values.

Due to the threshold dependency, we alternatively used the logistic transformation in the acceptance probability calculation. Although this transformation still requires a tuning parameter, CC, we demonstrated that EPD can accurately estimate the underlying distributions of various differential equations using CC values in the range of 100 to 300 (Comment 2).

We sincerely appreciate the reviewer for highlighting this point. We have added the rationale for using the logistic transformation in the Discussion section as below (line 250):

"...We also utilized the logistic transformation in the acceptance probability. While existing methods (such as ABC) use a threshold for the final decision, this can result in the shape of estimated parameter distribution varying depending on the threshold value (Fig. S6). To address this issue, we used the logistic transform, allowing the final decision to be made based on a random number between 0 and 1, instead of relying on a threshold. Following this transformation, the resulting value can consistently be used as the acceptance probability for any given dataset...."

4. The EPD method shows promise in the models evaluated, but its applicability to other types of differential equations and systems is not explored.

Answer: As reviewer's comment, we agree that validating the efficiency and robustness of the EPD method requires testing across various differential equations and systems. In our work, we initially validated the EPD using the exponential model, primarily to demonstrate the limitations of existing methods with the RCS dataset. We then applied the EPD to the logistic model and the target cell-limited model, both of which are supported by real-world datasets.. To show the wide applicability of EPD, We employ two additional famous equations: Michaelis-Menten equation [carson2013modelling, chapter 8] and Lorenz system [lorenz1963deterministic] (Figs 7-8).

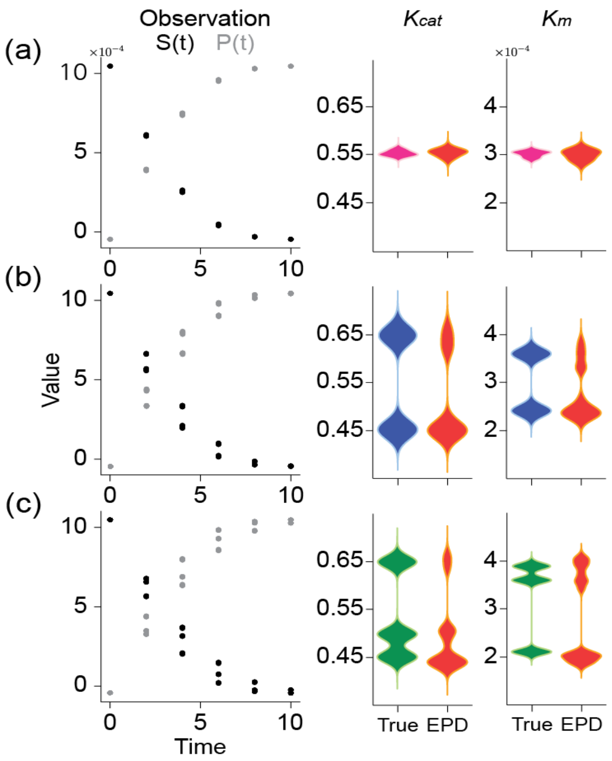

Fig 6. Estimation of parameter distribution for the Michaelis-Menten equation. Using a numerical solver, we generated RCS data from 1000 solutions of the Michaelis-Menten equation (left, $S(t)$, and $P(t)$). We then estimated the distributions of K_{cat} and K_m through EPD (right). For the unimodal (a), bimodal (b), and trimodal (c) scenarios, we found that EPD accurately captured the underlying parameter distributions.

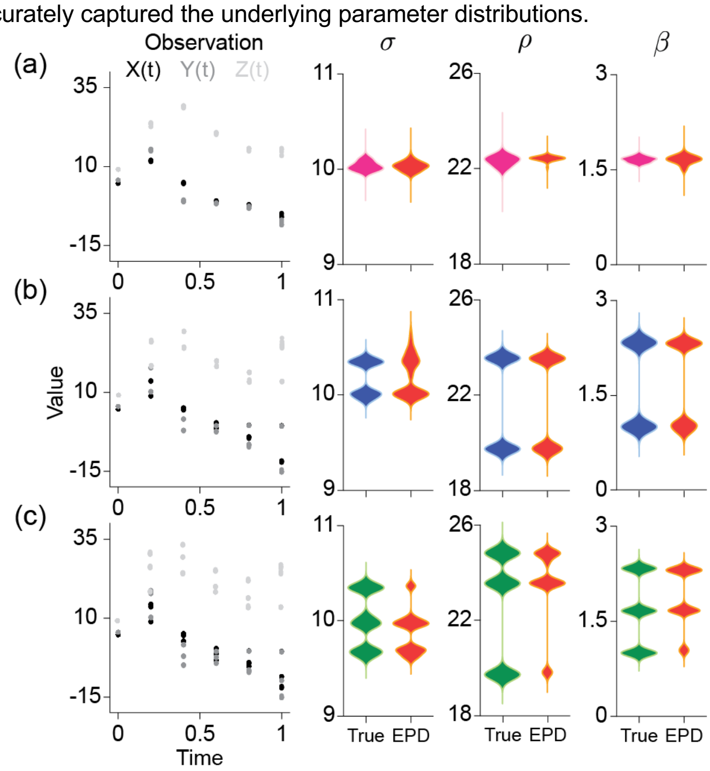

Fig 7. Estimation of parameter distribution for the Lorenz system. Using a numerical solver, we generated RCS data from 1000 solutions of the Lorenz equation (left, $X(t)$, $Y(t)$, and $Z(t)$). We then estimated the distributions of σ, ρ, and β through EPD (right). For the unimodal (a), bimodal (b), and trimodal (c) scenarios, we found that EPD accurately captured the underlying parameter distributions.

We have demonstrated that the EPD method can accurately estimate the given parameter distribution in other types as well. Further details have been added to the new subsections in the Results section.

References
[lorenz1963deterministic] Lorenz, E. N. (1963). Deterministic nonperiodic flow. Journal of atmospheric sciences, 20(2), 130-141.
[carson2013modelling] Carson, E., & Cobelli, C. (2013). Modelling methodology for physiology and medicine. Newnes.

Reviewer #3: This manuscript seeks to address a problem in parameter estimation when working with discrete time series data, where individual measurements are available, but not individual histories. The authors have proposed a novel method to estimate parameters in dynamical systems models of these data. However, I cannot recommend it for publication on this journal, primarily because the method proposed needs to be more fully developed to make a convincing case. I also have several concerns/suggestions for the authors that I hope will be helpful:

1. A major concern is that the authors only compare their method in one case to GP method. However, if the goal is to arrive at probability distributions of model parameters, then the method should also be compared with other Bayesian Estimation methods eg Metropolis Hastings. For which, priors could be multimodal, possibly circumventing the issues faced by GP in capturing true multimodal distributions.

Answer: We sincerely thank the reviewer for pointing out the lack of comparison model and for suggesting additional methods. We initially compared the estimation performance of EPD with that of GP. Through this revision, we have added estimation results with two additional methods, Approximate Bayesian Computation (ABC) and Metropolis-Hastings (MH) in Figure 1:

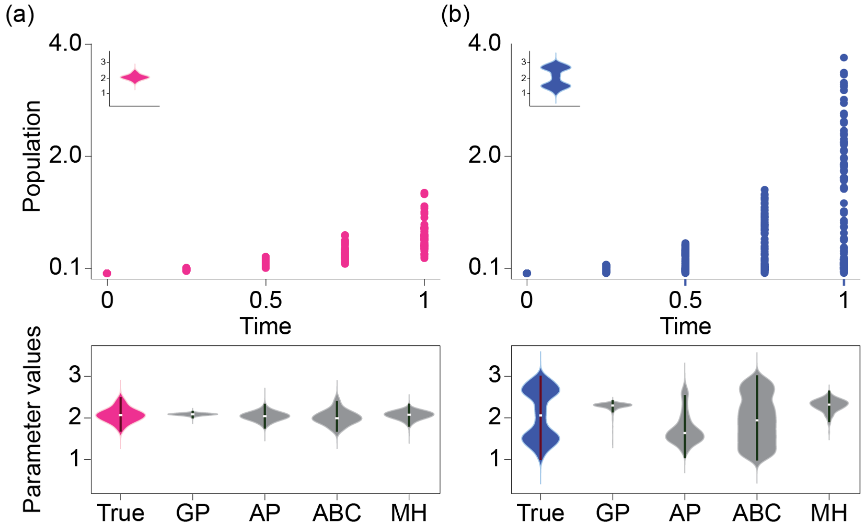

Fig 1. Parameter estimation in the exponential growth model with Repeated Cross-Sectional (RCS) data. An exponential growth model $y'(t) = ay(t)$ represents the amount of population, $y(t)$, changes over time, t. We then estimated parameter a that can fit the model to a given RCS data (a-b). When the true parameter distribution of a is unimodal (a, top-panel), corresponding RCS data is generated by parameters a, and populations per time do not diverge (a, top). In this case, previous methods, such as Gaussian Process (GP), All Possible combinations (AP), Approximate Bayesian Computation (ABC), and Metropolis-Hastings (MH), can estimate true parameter distributions (bottom) (a, bottom). When the true parameter distribution of a is bimodal (b, top-penal), populations per time diverge (b, top). In this case, previous methods fail to estimate the shape of true parameters (b, bottom). Additionally, we performed the same estimation task with various prior distributions and observed that both ABC and MH still failed to accurately estimate the true distribution (Fig. S1).

Specifically, we implemented the MH algorithm using the likelihood based on a normal distribution. The likelihood of the RCS data was computed as the product of the individual likelihoods for each data point in the dataset. However, the MH algorithm yields a distribution of parameter estimates with a single

peak, regardless of the varying shapes of the prior distribution. That is, MH fails to estimate parameter distribution when true parameter distribution is multimodal (See also Fig. S1):

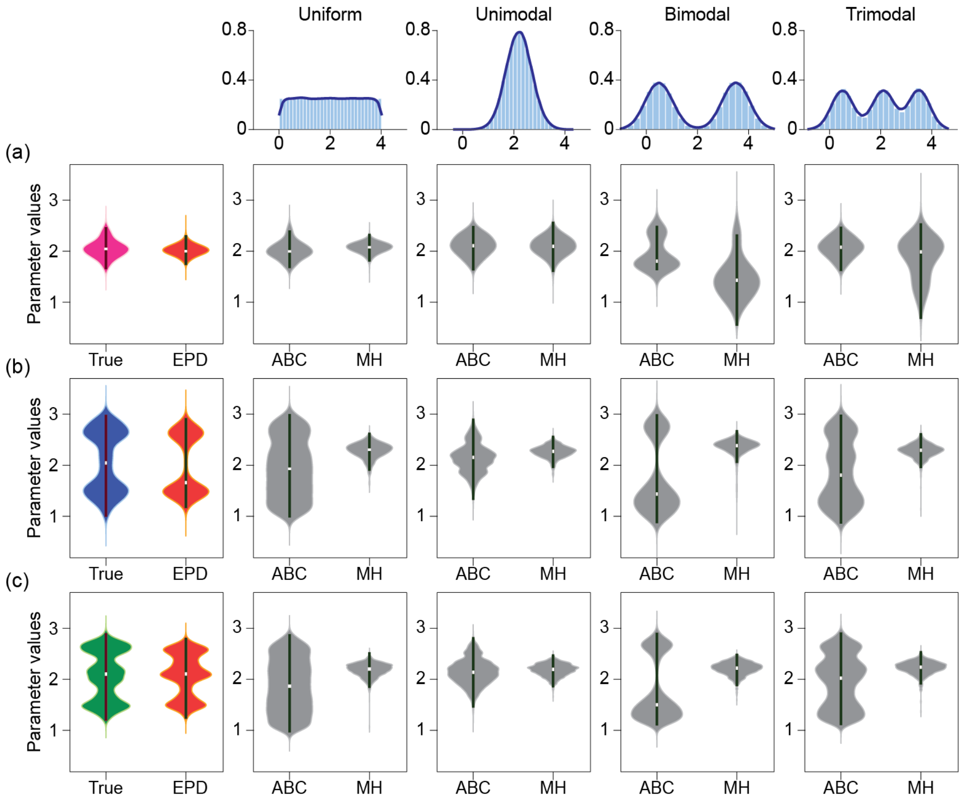

Fig S1. Parameter estimation results for the Exponential model using Approximate Bayesian Computation (ABC) and Metropolis-Hastings (MH), based on different shapes of the prior distribution (uniform, unimodal, bimodal, and trimodal). We estimate three True parameter distributions (unimodal (True-red), bimodal (True-blue), and trimodal (True-green)) corresponding to each dataset in Fig. 1. Without any assumptions about the prior distribution, EPD can accurately estimate the True parameter distributions (EPD-orange). (a-c). When the True parameter distribution is unimodal, all estimation results closely approximate the true distribution, except for ABC with a bimodal prior distribution (a). fHowever, when the true distribution is bimodal, all estimation results using ABC and MH show different patterns from the true distribution, except for ABC with a bimodal prior distribution (b). Similar to (b), when the true distribution is trimodal, all estimation results using ABC and MH show different patterns from the true distribution, except for ABC with a trimodal prior distribution (c). These results indicate that the ABC method estimates the similar shape of parameter distribution to the prior distribution, leading to inaccurate parameter estimates with inaccurate choice of prior distributions.

Similarly, ABC also fails to estimate the true distribution except when the prior distribution is similar to the true distribution.

It is well-established that the estimation of parameter distribution can vary depending on the shape of the prior distribution [swigon2019importance]. In classical Bayesian-based estimation methods like MH or ABC, the choice of prior significantly influences the estimated distribution, especially when dealing with RCS data. Unlike these methods, our approach (EPD) allows estimation without the researcher's subjectivity in selecting the prior. To avoid the bias introduced by a subjectively chosen prior, we automatically derived the prior distribution directly from the observed data. We have included this explanation in the Introduction and Methods sections of the manuscript (lines 45 and 343). Again, we thank the reviewer for highlighting this important aspect.

References
[swigon2019importance] Swigon, D., Stanhope, S. R., Zenker, S., & Rubin, J. E. (2019). On the importance of the Jacobian determinant in parameter inference for random parameter and random measurement error models. SIAM/ASA Journal on Uncertainty Quantification, 7(3), 975-1006.

2. The test cases, eg Fig 1, 2, etc are very artificial. I understand that these are demonstrations that the method proposed can successfully recover the true multimodal distributions on model parameters, but equally, a modeler applying classic methods to these data would notice that the data is not from a single but from multimodal distributions on model parameters and presumably account for that before curve fitting.

Answer: We agree with the author's comment that the several examples in this work are artificial. For example (exponential model in Fig. 1), we aimed to highlight the issues with traditional estimation methods for handling RCS data. As mentioned in the comments, this example also allows for grouping based on the researcher's subjectivity. Therefore, we have increased the number of RCS data in the original Figure 1 to make the researcher's subjectivity more difficult as below:

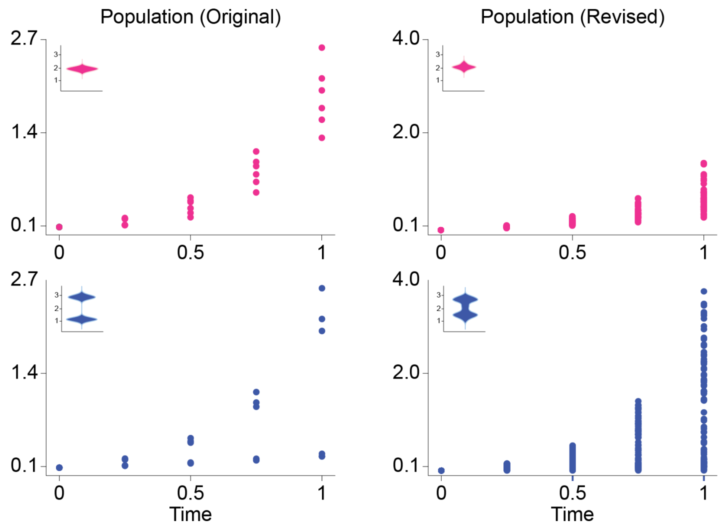

Fig. Comparison between the original RCS data used in the exponential model (left - top, bottom) and the newly revised RCS data (right - top, bottom). The original RCS data was obtained from 12 time trajectories (left), while the revised version was generated from 50 time trajectories (right). After this change, we can no longer separate the RCS data based on the researcher's subjectivity.

Following the modification of the data example, the corresponding changes in EPD results are shown below (Fig. 3).

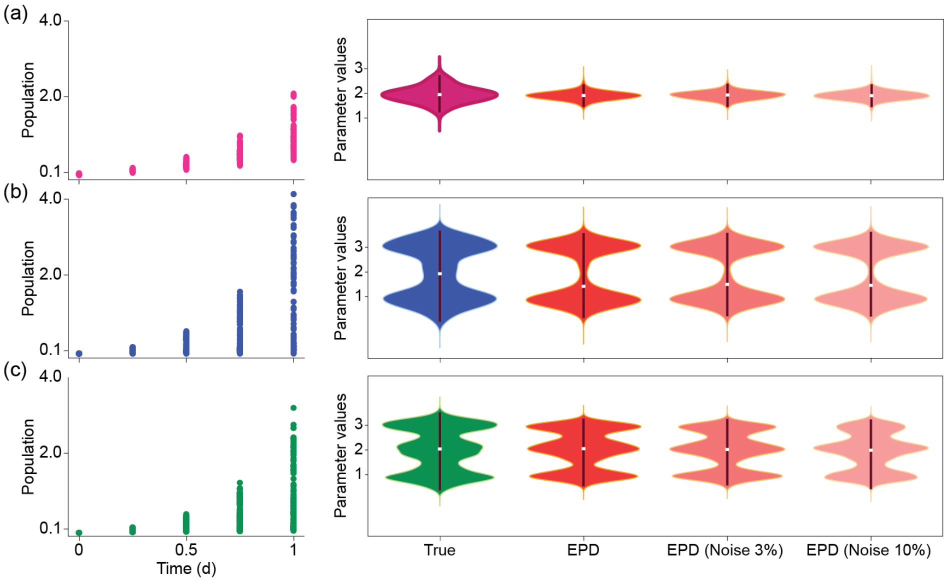

Fig 3.Estimation of true distributions by EPD in datasets exhibiting unimodal, bimodal, and trimodal parameter distributions within the Exponential Growth Model. (a) When the true parameter distribution is unimodal, we applied EPD on the observed data (left) and estimated the parameters (right). Notably, EPD accurately estimated parameter distributions even when we added 3% or 10% multiplicative noise to the data (b, c). Likewise, EPD was confirmed to estimate bimodal and trimodal parameter distributions effectively.

We have also updated all estimation results in our research (Figs. 4-10). Furthermore, in the original manuscript, we also included results from two cases of real-world experimental data: 1) biomarkers for diagnosing dementia [yada2023few], and 2) BALB/cJ mice infected with the influenza H1N1 virus [smith2018influenza]. In the first example, as the reviewer' comment, the data was pre-divided into two groups based on the amino acid length used by the researcher. However, any additional heterogeneity in the data was unknown, so the analysis was limited to estimate the parameters with the mean time trajectories over time t. In the second case, the dataset is four-dimensional, and there are six parameters to estimate. In this scenario, it is not intuitive for the modeler to manually divide the data into several groups. Therefore, the primary purpose of EPD is to automatically handle this type of complexity in data segmentation.

References
[yada2023few] Yada, Y., & Naoki, H. (2023). Few-shot prediction of amyloid β accumulation from mainly unpaired data on biomarker candidates. NPJ Systems Biology and Applications, 9(1), 59.
[smith2018influenza] Smith, A. P., Moquin, D. J., Bernhauerova, V., & Smith, A. M. (2018). Influenza virus infection model with density dependence supports biphasic viral decay. Frontiers in microbiology, 9, 1554.

3. The fits in Figure 4, panel (c) right box show a number of parameter combinations that were estimated but are very far from true parameter estimates. Given that the model is the logistic model, and hence very simple, it is concerning that the proposed method can have a high number of false positives that are such big outliers.

Answer: Thank you for pointing out the important issue. First, our data was based on assumptions corresponding to two biological scenarios: One with a low growth rate and high capacity, and the other with a high growth rate and low capacity. In the estimation results through EPD, we identified not only

the true two cases but also a new group with a low growth rate and low capacity. As the reviewer's comment, it can be considered as false positives. However, this occurred because the RCS data lost all continuous information about growth and capacity. Therefore, this can lead to all possible estimation outcomes, including both true and false positives, as identifying each subject corresponding to a data point is impossible. As a result, when we visualize the estimates using marginal distributions, we confirm that no additional information is provided. To verify the simulation result, we have drawn the trajectories corresponding to each parameter estimate as below:

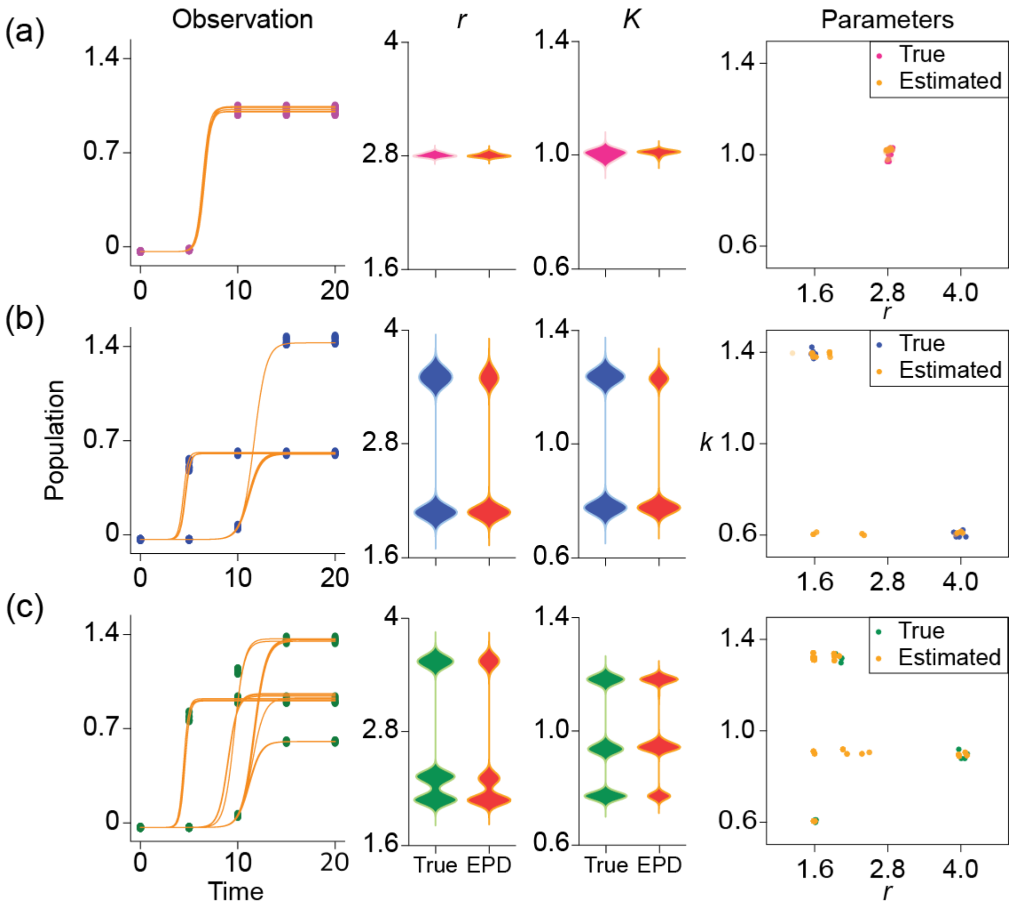

Fig 4. Estimation of parameter distribution for the logistic growth model. The model includes two parameters, r and k. For scenarios involving unimodal, bimodal, and trimodal distributions, we used the RCS data (left, Observation-dots) and compared true and estimated the parameter distributions (right, r, k-True, EPD) (a-c). EPD accurately estimates the true parameter distribution when the true distribution is unimodal (a). In the bimodal case, EPD also accurately captures the marginal distributions of r and k. The true parameter distribution in this case consisted of one group with a low growth rate and high capacity, and another group with a high growth rate and low capacity (b-Joint distribution, True). Interestingly, EPD not only identified these two true groups but also detected a new group with a low growth rate and low capacity (b-Joint distribution, Estimated), revealing that this problem is not fully identifiable (b). For the trimodal case, EPD accurately estimates the distributions (c). Similar to (b), we found the other near $r = 2.2$ and $K = 0.9$ (c-Joint distribution, Estimated). To verify that the estimated parameters are reasonable, we plotted all trajectories corresponding to the estimated parameters (Observation-yellow lines). This indicates that our estimation captured all parameters that pass through the RCS data. In other words, although they may not be included in the actual joint distribution, this indicates that we have successfully recovered all potential characteristics of the data.

Through the trajectory reconstruction, we found that the corresponding parameter estimates still penetrate all RCS data samples, indicating the identifiability problems in the RCS dataset. Thank you for pointing it out again, we have added this issue in the Discussion section (line 287).

"...we found the identifiable problem through the logistic example (Fig. 4). In this example, data is based on assumptions corresponding to two biological scenarios: one with a low growth rate and high capacity and the other with a high growth rate and low capacity. In the estimation results obtained through EPD, we identified not only the two true cases but also a new group with low growth rate and capacity (Fig. 4, Joint distribution). This is natural because the RCS data lost all continuous information regarding growth and capacity, leading to possible outcomes. Although we have presented only the raw estimation results to understand the RCS data, this issue can be resolved in actual estimations by adding constraints that prevent biologically implausible outcomes"

4. The parameter estimates in Fig 8 and 9 are very scattered. Is it reasonable to expect that the biological data being fitted can admit such diverse parameter values? This points to issues of parameter identifiability, and something that a modeler would attempt to address before undertaking the estimation itself. Also, line 171 on Page 11 seems to have a typo, the parameter names do not match those on Figure 9.

Answer: We agree with the reviewer's comment regarding the importance of addressing these issues (as mentioned in our third comment). However, the overall ranges of the parameter estimates in Figs. 10–11 (previously Figs. 8–9 in the initial manuscript) are consistent with those reported in previous studies [yada2023few, smith2018influenza]. In those studies, it was assumed that all parameters derived from experimental data follow a normal distribution. While this normality assumption may not capture the exact shape of the parameter distribution, it provides useful insight into the potential ranges (see Fig. S1). In contrast, the EPD method identifies plausible parameter pairs within these suggested ranges. As the reviewer's comment, we believe that applying suitable assumptions (constraints) to the parameter estimates could lead to more refined and accurate parameter distribution estimates. However, due to the lack of domain knowledge, we were unable to impose additional constraints, as the original papers [yada2023few, smith2018influenza] did not provide them.

Also, we have fixed typos on Page 11 (line 136) and the parameter names (Fig S3).

References
[yada2023few] Yada, Y., & Naoki, H. (2023). Few-shot prediction of amyloid β accumulation from mainly unpaired data on biomarker candidates. NPJ Systems Biology and Applications, 9(1), 59.
[smith2018influenza] Smith, A. P., Moquin, D. J., Bernhauerova, V., & Smith, A. M. (2018). Influenza virus infection model with density dependence supports biphasic viral decay. Frontiers in microbiology, 9, 1554.
[jo448density] Jo, H., Hong, H., Hwang, H. J., Chang, W., & Kim, J. K. Density Physics-Informed Neural Network reveals sources of cell heterogeneity in signal. cell cycle, 448, 947-951.

5. On line 7, the authors say "data is collected over time measuring the same 7 variables with different samples or populations at each time point", yet in methods, line 220, they say "Each Yi includes J observed data points at time ti". This is confusing and needs to be clarified. Were the number of observations constant at each time point or did these change? If they change, then sampling from the data points at each time ti can introduce a bias becase sampling from fewer observations will over emphasize their importance relative to data at time points with more observations

Answer: Thank you for pointing it out. We have replaced the index J (the number of observations at t_i) with J_i as it does not have to be a constant. Moreover, we have rewritten the Materials and Methods section for clarity.

As the reviewer mentioned, we also agree that the fewer observations at t_i make the bias in parameter estimates. To verify this, we conducted additional experiments to observe how the shapes of the parameter distributions obtained from EPD change when some of the observed data are missing in the exponential model (Fig. S3):

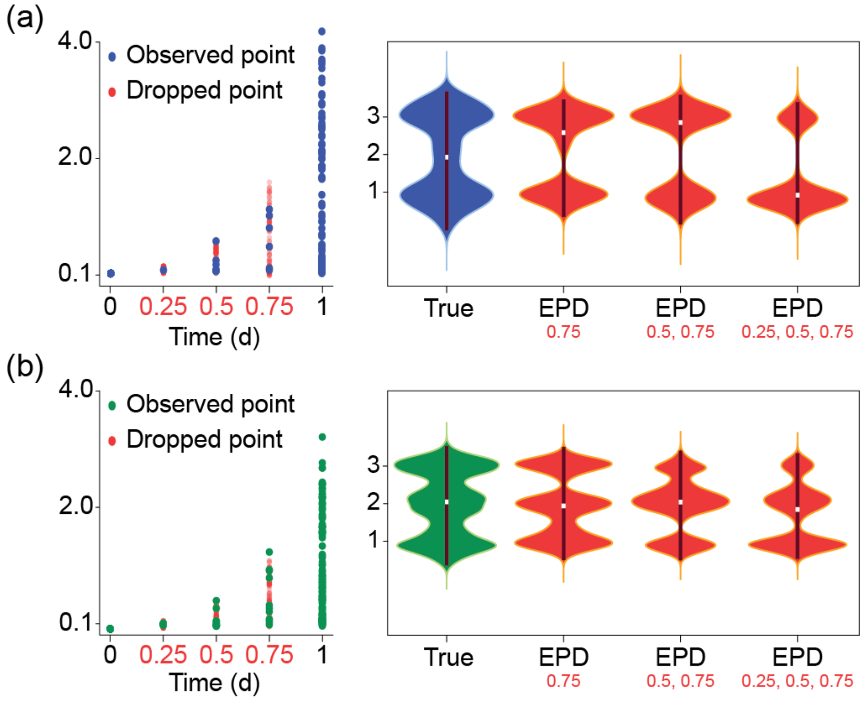

Fig S7. We estimate the parameter distributions using RCS data from Fig. 3(b-c, left) after removing 90% of the observation data points in three scenarios: 1) removal of data at $t = 0.75$, 2) removal of data at $t = 0.5$, and $t = 0.75$, and 3) removal of data at $t = 0.25$, $t = 0.5$, and $t = 0.75$ (Fig. S3, left - Dropped points). (a) When the underlying parameter distribution is bimodal (right, True), EPD accurately estimates the True parameter distribution (EPD 0.75) using RCS data after removing 90% of the observation data points at $t = 0.75$. Similarly, when 90% is removed at $t = 0.5$ and $t = 0.75$, the estimation using EPD remains close to the underlying distribution (EPD 0.5, 0.75). However, when 90% is removed at $t = 0.25$, $t = 0.5$, and $t = 0.75$, a bias is observed in the height of the peak (EPD 0.25, 0.5, 0.75). (b) When the underlying parameter distribution is trimodal (right, True), EPD can estimate the underlying distribution using RCS data after removing 90% of the data points at $t = 0.75$ (right, EPD 0.75). Unlike the bimodal case (a), the peak heights differ from the True distribution in two cases: 1) when observation data points are removed at $t = 0.5$ and $t = 0.75$ (right, EPD 0.5, 0.75), and 2) when points are removed at $t = 0.25$, $t = 0.5$, and $t = 0.75$ (right, EPD 0.25, 0.5, 0.75). Notably, although the missing data yields the bias in the heights, EPD can still estimate the shape of the parameter distribution, stemming from the heterogeneity of the data.

Specifically, we removed 90% of the observation data points in Fig. 3(a-c, left) with three scenarios: 1) at $t = 0.75$, 2) at $t = 0.5$ and $t = 0.75$, and 3) at $t = 0.25$, $t = 0.5$, and $t = 0.75$ (Fig. S7, left - Dropped points). As the number of missing data points increased, the estimation results using EPD showed a bias, resulting in different heights of the peaks (Fig. S7, right).

We also attached a detailed explanation in the Discussion section as below (line 266):

"...we observed a bias due to the missing observation data. To investigate this further, we conducted additional experiments to examine how the shapes of the parameter distributions obtained from EPD are affected by the

missing data in the exponential model (Fig. S7(a-c), left). In particular, we removed 90\% of the observation data at specific time points (Fig. S7, left - Dropped points). As the amount of missing data increased, the estimation results using EPD exhibited a bias, leading to variations in peak heights (Fig. S7, right). Notably, although the missing data introduces bias in the peak heights, EPD still estimates the overall shape of the parameter distribution, reflecting the heterogeneity of the RCS data.….”

We sincerely thank the reviewer for highlighting this important issue.

6. How big should N be (line 226), ie, how many artificial trajectories should be generated from the data? How does this depend on the number of data points, the number of model parameters, the variance in the data etc etc?

Answer: Thank you for pointing it out. The required number of artificial trajectories is proportional to the product of data points at each time point (line 328). It is also proportional to the number of model parameters and equations. We have attached the below table, including the number of trajectories and its computational cost simultaneously in the Methods and Materials section (p17):

Equation	Parameter	n_y	n_p	T	J_i	N	Running Time (sec)
Exponential model	Unimodal	1	1	5	50	1000	4.06
	Bimodal				100		4.18
	Trimodal				150		4.26
Exponential Model w noise	Unimodal	1	1	5	50	10000	80.75
	Bimodal				100		83.62
	Trimodal				150		84.18
Logistic Model	Unimodal	1	2	5	12	3000	162.70
	Bimodal				12		181.54
	Trimodal				12		202.69
	Real-world				12-13		188.85
Target Cell Limited model	Unimodal	4	6	13	12	1000	4555.18
	Bimodal				12		9036.38
	Trimodal				12		12318.30
	Real-World				10	10000	111667.74
Lorentz System	Unimodal	3	3	6	12	1000	28.90
	Bimodal				24		39.09
	Trimodal				36		42.62
Michaelis -Menten	Unimodal	2	2	6	12	1000	9.50
	Bimodal				24		11.39
	Trimodal				36		10.80

Table 3. Computational Cost for the EPD corresponding to each equation and dataset. The running time is measured based on several factors, including the number of equations (n_y), the number of parameters (n_p), the number of time points (T), the number of data points for each time point, (J_i), the total number of data points (N), and the number of artificial trajectories (N). The results indicate that the computational cost is directly proportional to n_y, n_p, T, and N, while showing a small correlation with the underlying parameter distribution.

7. Equation (3), why this objective function? What if we minimize simply sum of squares? Or employ some other minimization scheme?

Answer: Initially, we attempted to utilize the sum of squares error. However, we found two important issues with the sum of squares error. First, the varying scale of magnitude in observation points over time t introduces errors in the target cell limited model (Fig. S4):

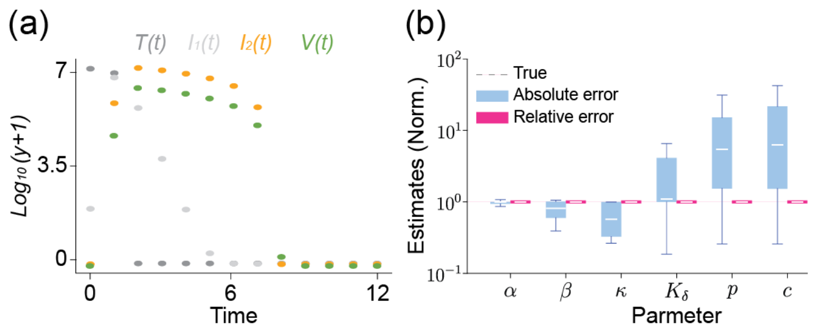

Fig S4. Comparison of estimation results using EPD with two different objective functions: Absolute error and relative Error (Ours) in a Target Cell-Limited Model (a) (a) We generated trajectories for $T(t), I_1(t)\,, I_2(t)$, and $V(t)$ corresponding to one set of six parameters $(p, K_\delta, \beta, c, \kappa, \delta) = (2.4 \times 10^{-4}, 1.6, 13.0, 4.0, 1.6 \times 10^6, 4.5 \times 10^5)$ within the target cell-limited model. (b) We compared the parameter estimates obtained using the absolute error (i.e., $L_n(\mathbf{p}) = \sum_{i=1}^{T} |\bar{\mathbf{y}}_n(t_i; \mathbf{p}) - \tilde{\mathbf{y}}_n(t_i)|^2$), to those obtained using the relative error $L_n(\mathbf{p})$ in (Eq.(4)) (Blue and red box plots, respectively). The box plot results reveal that the absolute error introduces biases in the parameter estimates compared to the relative error.

In this example, progression rate κ and death rate δ are mainly determined by the period when the concentration of infected sell I_2 reached the highest value (i.e., $I_2 \sim 10^7$ in Fig. S4(a)-I_2). In contrast, the parameters p and c are mainly affected by the balance between virus production and clearance, especially during the low viral load stages. (i.e., $I_2 = 0$ and $V \sim 0$). Therefore, Optimization steps for finding the minimizer of the $L(\mathbf{p})$ are mainly conducted by finding the suitable κ and δ as the corresponding absolute error terms in $L(\mathbf{p})$ are dominant (Fig. S4(b)).

Next, we also found that the bias in the values of $L(\mathbf{p})$ in the EPD algorithm, stemming from the absolute size of the observation data point for a fixed time t. This leads to biased parameter estimates. To verify this, we calculate $L(\mathbf{p}_n)$, for $n = 1, \ldots, N$ with sum of squares, then divided the distribution of $L(\mathbf{p}_n)$ values into two groups: one group (blue) selecting the smallest observation at the final time point ($t = 1$) and the other group (red) selecting the largest observation (Fig. S5):

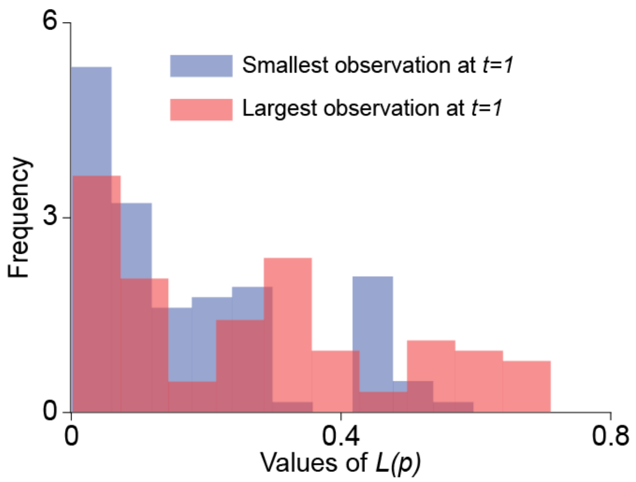

Fig S5. Distribution of L(p) with an absolute error when a single observation is randomly sampled at each time point, with the smallest observation (blue) and the largest observation (red) selected at $t = 1$. The distribution of $L(p)$ varies according to the magnitude of the observations. As the $L(p)$ distribution associated with the larger observation tends to have higher overall values, the likelihood of it being ultimately selected decreases.

As a result, the parameter estimates from the red graph, which have relatively larger values of $L(\mathbf{p})$, exhibit higher rejection probabilities, leading to a reduction in the magnitude of the corresponding peak in the estimated parameter distribution. To avoid this bias, we have changed the measurement in $L(\mathbf{p})$ to the "relative" error.

We also have added these issues to the Discussion section (line 225). We sincerely thank the reviewer for pointing it out.

8. Is there a theoretical basis for the definition of an (line 249)? The authors themselves point to challenges with selecting 'c' which seems ad hoc at present. Is there any guidance on how to choose c? what about other forms for an?

Answer: We could not find the theoretical guidance for the choice of the scaling factor c, as it is kind of a hyperparameter (such as a threshold of rejection sampling in the ABC method). However, selecting an appropriate scaling factor is important for estimating the shape of parameter distributions. To explore this issue, we additionally have revised Fig. 10 to emphasize how the estimation results can vary depending on values of the scaling factor:

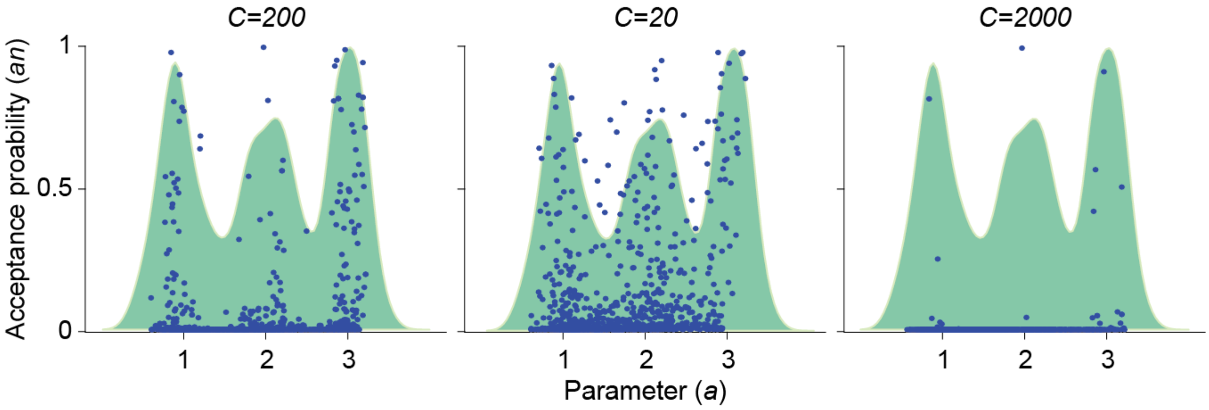

Fig 10. Distribution of the acceptance probability a_n with different scaling factors C in the exponential model with a trimodal true parameter distribution (green). We first calculate the parameter $\mathbf{p}_n$ corresponding to n-th artificial trajectory $\mathbf{y}_n$ (Fig. 2(b), left), then plotted $(\mathbf{p}_n, a_n)$ (blue dots), for $n = 1, \ldots, N$. When $C = 200$, only high values a_n are located near the peaks of the true parameter distribution (green), leading to correct parameter estimates (left). However, when C is too small ($C = 20$), high values of a_n are widely spread across the entire parameter range (middle). Conversely, when C is too large ($C = 2000$), the overall acceptance probabilities are concentrated near 0, resulting in poor parameter estimates (right).

Although there is no theoretical guideline for selecting C, one promising approach is to generate simulation data with a range similar to the actual data across various distributions in advance, and then determine the appropriate range of C that can estimate the distribution. In our simulation dataset, we selected C in the range of 100 to 300 across the five different types of equations (Table 2):

Type	Exponential	Logistic	Target Cell Limited Model	Lorentz	Michaelis Menten
Unimodal	200	100	300	100	300
Bimodal	200	100	300	100	300
Trimodal	200	200	300	300	300
Real	-	200	300	-	-

Table 2. Scaling factors for the EPD corresponding to each equation and dataset. The scaling factor C was selected within the range of 100 to 300 across the five different types of equation, to estimate distribution that closely matches the true distribution. For the real-world dataset, the scaling factor was determined based on the most prevalent value observed in the simulation datasets.

Through simulation tests with the above scaling factor values in the range of 100 to 300, we have shown that EPD can accurately estimate the shape of true parameter distributions throughout various five equations with different types of RCS datasets. We now have added information of the scaling factor in Materials and Methods (p17).

As we mentioned, the definition of a_n in EPD was developed inspired by the Approximate Bayesian Computation (ABC) method. In the early stages of development, we attempted to directly apply the rejection algorithm in the ABC method (i.e., we accept parameter $\mathbf{p}$ if $L(\mathbf{p}) < \varepsilon$). However, the shape of the parameter estimates were too sensitive depending on the value of \varepsilon (see Fig S6 below), especially when the true parameter distribution has several peaks.

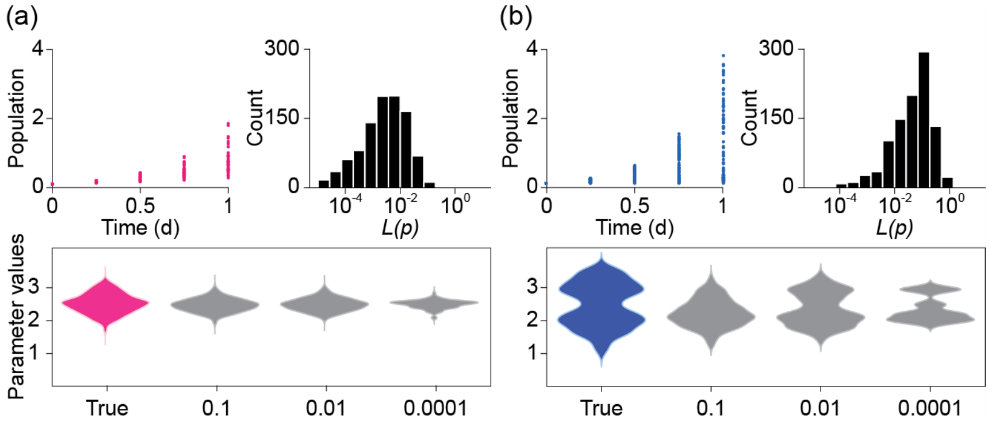

Fig S6. Estimation of the underlying parameter distribution of the Exponential model using EPD with relative error. We applied rejection sampling that is typically used in the ABC method. (a-b). When the shape of the underlying distribution is unimodal (True), $L(p)$ with relative error in EPD estimation is not significantly affected by the threshold values (0.1, 0.01, 0.0001) (a). Conversely, if the underlying distribution is bimodal (True), the estimated shape can differ from the underlying distribution depending on the threshold values (b).

Due to the high threshold dependency, we alternatively used the logistic transformation in the acceptance probability calculation. Although this transformation still requires a scaling factor, we experimentally demonstrated that a scaling factor in the range of 100 to 300 is sufficiently applicable

through 15 simulations (three different types of true distributions × five different systems). We also have added an issue on the choice of scaling factors in Discussion section (line 247 & 262):

"...We also utilized the logistic transformation in the acceptance probability. While existing methods (such as ABC) use a threshold for the final decision, this can result in the shape of estimated parameter distribution varying depending on the threshold value (Fig. S64). To address this issue, we used the logistic transform, allowing the final decision to be made based on a random number between 0 and 1, instead of relying on a threshold. Following this transformation, the resulting value can consistently be used as the acceptance probability for any given dataset...."

"...Although careful selection of the scaling factor is required, EPD can accurately estimate the underlying distributions across various differential equations using scaling factor values between 100 to 300 (Table 2)...."

We sincerely thank the reviewer for pointing it out.

9. What about estimation multiple data time courses simultaneously? So if a model has 2+ equations an we had measurements of 2+ variables, then how would this method be amended, or how would it perform?

Answer: Thank you for pointing it out. As the reviewer's comment, we originally had only one complex example (the target cell-limited model; four equations and six parameters). To demonstrate the applicability of EPD to more complex models, we have added two more cases: the Michaelis-Menten equation [carson2013modelling] and the Lorenz system [lorenz1963deterministic]. The Michaelis-Menten (Lorenz system) has three (three) equations and two (three) parameters, respectively.

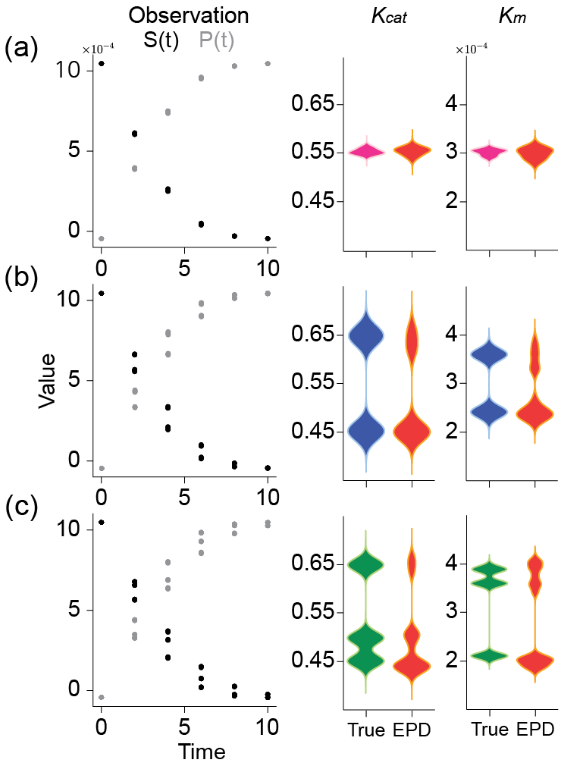

Fig 6. Estimation of parameter distribution for the Michaelis-Menten equation. Using a numerical solver, we generated RCS data from 1000 solutions of the Michaelis-Menten equation (left, $S(t)$, and $P(t)$). We then estimated the distributions of K_{cat} and K_m through EPD (right). For the unimodal (a), bimodal (b), and trimodal (c) scenarios, we found that EPD accurately captured the underlying parameter distributions.

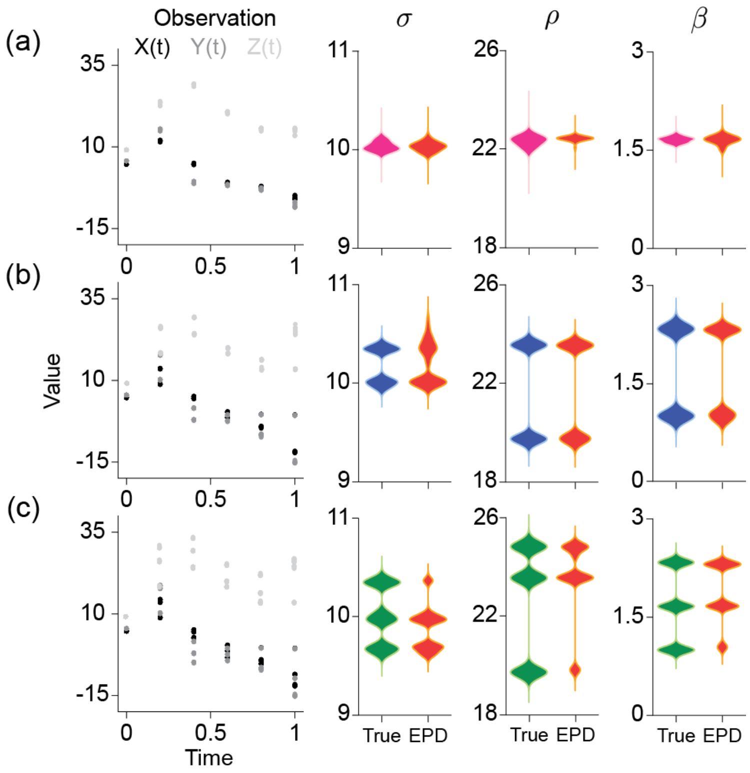

Fig 7. Estimation of parameter distribution for the Lorenz system. Using a numerical solver, we generated RCS data from 1000 solutions of the Lorenz equation (left, $X(t)$, $Y(t)$, and $Z(t)$). We then estimated the distributions of σ, ρ, and β through EPD (right). For the unimodal (a), bimodal (b), and trimodal (c) scenarios, we found that EPD accurately captured the underlying parameter distributions.

Through new examples, we have demonstrated that the EPD method can accurately estimate the given parameter distribution in other types as well. Further details have been added to the new subsections in the Results section.

References
[lorenz1963deterministic] Lorenz, E. N. (1963). Deterministic nonperiodic flow. Journal of atmospheric sciences, 20(2), 130-141.
[carson2013modelling] Carson, E., & Cobelli, C. (2013). Modelling methodology for physiology and medicine. Newnes.

10. The authors also hint at the method being computationally intensive and with the simplicity of the models they considered here, this can be potentially a bottleneck that may be undesirable.

Answer: As the reviewer pointed out, the computational costs for each artificial trajectory primarily increase due to the following two tasks: 1) solving the system using a numerical solver, and 2) fitting the solution through optimization techniques. For the first task, the computational time is proportional to the number of equations required to generate the artificial trajectory. For the second task, as the number of parameters (or the dimension of the parameter space) increases, a larger number of artificial trajectories is required to search the parameter space. Consequently, the computational cost increases proportional to the number of equations, parameters, and artificial trajectories.

To overcome this, as a future direction, we are considering the use of a machine learning-based model emulator (such as Physics-informed Neural Networks incorporating hypernetworks [de2021hyperpinn] or DeepONet [lu2021learning, hadorn2022shift, seidman2022nomad, lee2023hyperdeeponet]) to handle larger systems with RCS data more efficiently. The utilization of such emulators, which are pre-trained to approximate system solutions and optimization processes, is expected to significantly reduce the computational costs associated with these tasks.

We agree that this is a critical point for future exploration, and we have added the following limitation and future strategy to the Discussion section (line 277):

"...computational cost arises from when calculating the solution through a numerical solver and when fitting the solution through optimization techniques. As parameters were selected from a large set of synthetic trajectories that were obtained from the numerical solver, the computational costs became substantial, since it is proportional to both the complexity of equations and the number of trajectories. For future research, we are considering the use of a machine learning-based model emulator (such as Physics-informed Neural Networks incorporating hypernetworks [de2021hyperpinn] or DeepONet [lu2021learning, hadorn2022shift, seidman2022nomad, lee2023hyperdeeponet]) to handle larger systems with RCS data more efficiently. Specifically, the utilization of such emulators, which are pre-trained to approximate system solutions and optimization processes, is expected to significantly reduce the computational costs associated with these tasks."

references

[de2021hyperpinn] de Avila Belbute-Peres, F., Chen, Y. F., & Sha, F. (2021). HyperPINN: Learning parameterized differential equations with physics-informed hypernetworks. The symbiosis of deep learning and differential equations, 690.
[lu2021learning] Lu, L., Jin, P., Pang, G., Zhang, Z., & Karniadakis, G. E. (2021). Learning nonlinear operators via DeepONet based on the universal approximation theorem of operators. Nature machine intelligence, 3(3), 218-229.
[hadorn2022shift] Hadorn, P. S. (2022, March). Shift-deeponet: Extending deep operator networks for discontinuous output functions. ETH Zurich, Seminar for Applied Mathematics.
[seidman2022nomad] Seidman, J., Kissas, G., Perdikaris, P., & Pappas, G. J. (2022). NOMAD: Nonlinear manifold decoders for operator learning. Advances in Neural Information Processing Systems, 35, 5601-5613.
[lee2023hyperdeeponet] Lee, J. Y., Cho, S. W., & Hwang, H. J. (2023). HyperDeepONet: learning operator with complex target function space using the limited resources via hypernetwork. arXiv preprint arXiv:2312.15949.
